# Publisher Correction: Twisted moiré conductive thermal metasurface

**DOI:** 10.1038/s41467-024-47160-4

**Published:** 2024-04-03

**Authors:** Huagen Li, Dong Wang, Guoqiang Xu, Kaipeng Liu, Tan Zhang, Jiaxin Li, Guangming Tao, Shuihua Yang, Yanghua Lu, Run Hu, Shisheng Lin, Ying Li, Cheng-Wei Qiu

**Affiliations:** 1 Department of Electrical and Computer Engineering, National University of Singapore, Kent Ridge, 117583 Republic of Singapore; 2 State Key Laboratory of Extreme Photonics and Instrumentation, ZJU-Hangzhou Global Scientific and Technological Innovation Center, Zhejiang University, Hangzhou, 310027 China; 3 International Joint Innovation Center, Key Lab. of Advanced Micro/Nano Electronic Devices & Smart Systems of Zhejiang, The Electromagnetics Academy of Zhejiang University, Zhejiang University, Haining, 314400 China; 4 Wuhan National Laboratory for Optoelectronics and State Key Laboratory of Material Processing and Die and Mould Technology, School of Materials Science and Engineering, Huazhong University of Science and Technology, Wuhan, 430074 China; 5 Smart Materials for Architecture Research Lab, Innovation Center of Yangtze River Delta, Zhejiang University, Jiaxing, 314100 China; 6 State Key Laboratory of Coal Combustion, School of Energy and Power Engineering, Huazhong University of Science and Technology, Wuhan, 430074 China; 7 Chongqing 2D Materials Institute, Chongqing, 400015 China

**Keywords:** Metamaterials, Phase transitions and critical phenomena, Structural materials

Correction to: *Nature Communications* 10.1038/s41467-024-46247-2, published online 09 March 2024

The original version of this Article contained errors in the numbering sequence of the Equations (6) and (7). The correct numbering sequence of the Equations is (5) and (6). This has been corrected in the PDF and HTML versions of the Article.

