# Supplementary material for: Twisted moiré conductive thermal metasurface
Source: Nat Commun. 2024 Mar 9;15:2169. doi: 10.1038/s41467-024-46247-2 (PMC10924968; doi:10.1038/s41467-024-46247-2)
Supplement: Supplementary file 1 — Supplementary Information [file 41467_2024_46247_MOESM1_ESM.pdf]

Supplementary Information

Twisted Moiré Conductive Thermal Metasurface

Huagen Li,[1,8] Dong Wang,[2,3,8] Guoqiang Xu,[1] Kaipeng Liu,[1] Tan Zhang,[1] Jiaxin Li,[1] Guangming Tao,[4] Shuihua Yang,[1] Yanghua Lu,[5] Run Hu,[6] Shisheng Lin,[2,7] Ying Li,[2,3,*] and Cheng-Wei Qiu[1,*]

[1]*Department of Electrical and Computer Engineering, National University of Singapore, Kent Ridge 117583, Republic of Singapore*

[2]*State Key Laboratory of Extreme Photonics and Instrumentation, ZJU-Hangzhou Global Scientific and Technological Innovation Center, Zhejiang University, Hangzhou 310027, China*

[3]*International Joint Innovation Center, Key Lab. of Advanced Micro/Nano Electronic Devices & Smart Systems of Zhejiang, The Electromagnetics Academy of Zhejiang University, Zhejiang University, Haining 314400, China.*

[4]*Wuhan National Laboratory for Optoelectronics and State Key Laboratory of Material Processing and Die and Mould Technology, School of Materials Science and Engineering, Huazhong University of Science and Technology, Wuhan 430074, China.*

[5]*Smart Materials for Architecture Research Lab, Innovation Center of Yangtze River Delta, Zhejiang University, Jiaxing 314100, China*

[6]*State Key Laboratory of Coal Combustion, School of Energy and Power Engineering, Huazhong University of Science and Technology, Wuhan 430074, China.*

[7]*Chongqing 2D Materials Institute, Chongqing, 400015, P. R. China.*

[8]*These authors contributed equally: Huagen Li, Dong Wang*

[*]Email: eleying@zju.edu.cn; chengwei.qiu@nus.edu.sg

Supplementary Note 1: The determination of effective thermal conductivity along with the change of twisted angles

Firstly, as shown in Fig. S1, the background temperature should be a straight line along the y-axis if the effective thermal conductivity tensor κ_1^{eff} of the twisted stripes in a circular region is equivalent to the background thermal conductivity κ_{b}. Thus, in order to measure the effective thermal conductivity of the central twisted stripe region more accurately as we vary the value of κ_{b} to match the κ_1^{eff}, we introduce the total variance σ^2 of temperature changes along the line (x = 5cm) in the background region and let it to be far smaller than 0.01. The average temperature of all the points is denoted by $\overline{T_v}$ and the temperature value of the *nth* point is described by T_i. Therefore, the $\overline{T_v}$ and σ^2 can be derived as follows:

$$\overline{T_v} = \frac{\sum_{i=1}^{n} T_i}{n} \tag{S1}$$

$$\sigma^2 = \frac{\sum_{i=1}^{n}\left(T_i - \overline{T_v}\right)^2}{n} \ll 0.01 \tag{S2}$$

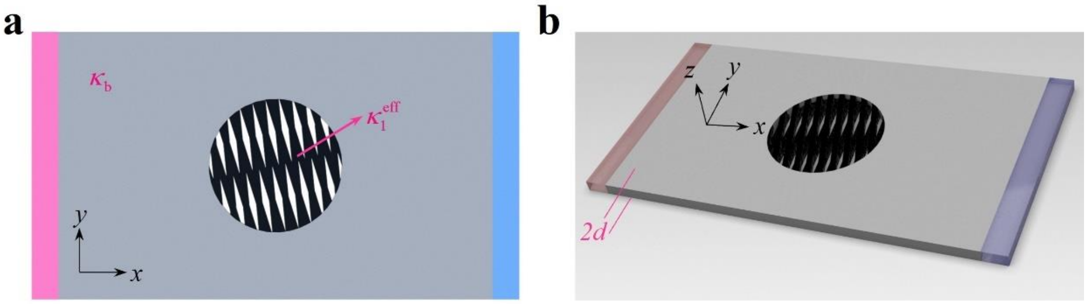

Fig. S1 Schematic diagram of the determination method of effective thermal conductivity. **a** 2D Schematic of the corresponding structure via this method. **b** 3D view of this structure.

Supplementary Note 2: Derivations of twisted thermotics theory in diffusive systems

As we all know, a crucial feature of the heat conduction equation is its form invariance under any coordinate transformation[1]. The heat conduction equation is shown as follows:

$$\rho c \frac{\partial T}{\partial t} = \kappa \nabla^2 T \tag{S3}$$

where T is temperature, ρ is the density, c is the specific heat capacity, and κ is the thermal conductivity tensor.

Under the coordinate transformation, the heat equation can be transformed into a similar form shown below:

$$\rho' c' \frac{\partial T}{\partial t} = \kappa' \nabla'^2 T \tag{S4}$$

Now we can solve the equation to acquire the κ'. Firstly, for physicists, by expressing the above equation in a curvilinear coordinate system (x^i and x^j, i = 1, . . ., n or j = 1, . . ., n) corresponding to a transformation, we have

$$\rho c \frac{\partial T}{\partial t} = \frac{\partial}{\partial x^i} \kappa^{ij} \frac{\partial}{\partial x^j} T + \Gamma^i_{ik} \kappa^{kj} \frac{\partial}{\partial x^j} T \tag{S5}$$

where Γ^i_{ik} is the Christoffel symbol satisfying the following equation:

$$\Gamma^i_{ik} = \frac{1}{2} g^{il} \frac{\partial}{\partial x^k} g_{il} = \det(J) \frac{\partial}{\partial x^k} \frac{1}{\det(J)} \tag{S6}$$

where g is the metric tensor, and J is the Jacobian matrix corresponding to the transformation.

To rewrite the above equation in the physical Cartesian coordinate system, we perform the variable changes from x^i and x^j to the Cartesian coordinate x'^i and x'^j and acquire

$$\frac{\rho c}{\det(J)} \frac{\partial T}{\partial t} = \rho c \frac{\partial \det^{-1}(J) T}{\partial t} = \frac{\partial}{\partial x'^i} \left[\frac{J \kappa J^T}{\det(J)} \right]^{ij} \frac{\partial}{\partial x'^j} T \tag{S7}$$

where J^T and $\det(J)$ are the transverse and the determinant of the Jacobian matrix J, respectively. We can see that the desired $\rho' c'$ and thermal conductivity κ' are as follows:

$$\kappa' = \frac{J \kappa J^T}{\det(J)} \tag{S8}$$

$$\rho' c' = \frac{\rho c}{\det(J)} \tag{S9}$$

For heat diffusion, we are mainly focused on the steady-state temperature field T that obeys Fourier's law[1]

$$\kappa \nabla^2 T = 0 \tag{S10}$$

Therefore, for the steady-state temperature fields, the potential thermal coupling mechanism might lie in the change of thermal conductivity tensor κ' with respect to the

original thermal conductivity tensor κ after the coordinate transformation. Now considering each stripe layer (upper or lower) with different thermal conductivities κ_{I} and κ_{II} which are composed of two kinds of stripe widths w_{I} and w_{II} (The subscripts $_{\mathrm{I}}$ and $_{\mathrm{II}}$ indicate the two kinds of stripe in either the upper or lower stripe layer, respectively), the anisotropic thermal conductivity tensors for both layers are then derived as follows[2,3]:

$$\kappa_{\text{upper}}^{\text{eff}} = \begin{pmatrix} \kappa_x & 0 \\ 0 & \kappa_y \end{pmatrix} = \begin{pmatrix} \frac{\left(w_{\mathrm{I}} + w_{\mathrm{II}}\right)\kappa_{\mathrm{I}}\kappa_{\mathrm{II}}}{w_{\mathrm{II}}\kappa_{\mathrm{I}} + w_{\mathrm{I}}\kappa_{\mathrm{II}}} & 0 \\ 0 & \frac{w_{\mathrm{I}}\kappa_{\mathrm{I}} + w_{\mathrm{II}}\kappa_{\mathrm{II}}}{w_{\mathrm{I}} + w_{\mathrm{II}}} \end{pmatrix} \tag{S11}$$

$$\kappa_{\text{lower}}^{\text{eff}} = \begin{pmatrix} \kappa_x & 0 \\ 0 & \kappa_y \end{pmatrix} = \begin{pmatrix} \frac{\left(w_{\mathrm{I}} + w_{\mathrm{II}}\right)\kappa_{\mathrm{I}}\kappa_{\mathrm{II}}}{w_{\mathrm{II}}\kappa_{\mathrm{I}} + w_{\mathrm{I}}\kappa_{\mathrm{II}}} & 0 \\ 0 & \frac{w_{\mathrm{I}}\kappa_{\mathrm{I}} + w_{\mathrm{II}}\kappa_{\mathrm{II}}}{w_{\mathrm{I}} + w_{\mathrm{II}}} \end{pmatrix} \tag{S12}$$

Then, assuming that the original angles of the upper and lower layers compared with the positive direction of y axis are both set as α and the direction of heat flux about the positive direction of x axis is $\varphi = 0$, the modified thermal conductivity based on its form invariance under coordinate transformation is then:

$$\kappa_{\text{upper}}^{\text{eff}}\Big|^{\alpha} = \frac{J_1 \kappa_{\text{upper}}^{\text{eff}} J_1^T}{\det(J_1)} = \begin{pmatrix} \kappa_x \cos^2(\alpha) + \kappa_y \sin^2(\alpha) & \left(-\kappa_x + \kappa_y\right)\cos(\alpha)\sin(\alpha) \\ \left(-\kappa_x + \kappa_y\right)\cos(\alpha)\sin(\alpha) & \kappa_x \sin^2(\alpha) + \kappa_y \cos^2(\alpha) \end{pmatrix} \tag{S13}$$

where J_1 is the Jacobian matrix of the coordinate transformation between the original vs. modified coordinate systems, J_1^T and $\det(J_1)$ are the transpose and the determinant of the J_1, respectively. Similarly, we can obtain the $\kappa_{\text{lower}}^{\text{eff}}\Big|^{\alpha}$ of upper layer which is the equal to that of upper layer due to the two layers with the same original angle α represented by the superscript $^{\alpha}$. The subscripts $_{\text{upper}}$ and $_{\text{lower}}$ indicate upper and lower layers, respectively.

According to the effective medium theory, the effective thermal conductivity tensor κ^{eff} can be achieved at the interface of the upper and lower layers after twisting the upper layer an angle θ_1 and the lower layer an angle θ_2 (The subscripts $_1$ and $_2$ indicate upper and lower layers, respectively), and we can obtain the corresponding equation in the Cartesian coordinate as follows:

$$\kappa^{\text{eff}}=\begin{pmatrix} \dfrac{\kappa_x\left[\cos^2(\theta_1-\alpha)+\cos^2(\theta_2-\alpha)\right]+\kappa_y\left[\sin^2(\theta_1-\alpha)+\sin^2(\theta_2-\alpha)\right]}{2} & \dfrac{(-\kappa_x+\kappa_y)\left[\cos(\theta_1-\alpha)\sin(\theta_1-\alpha)+\cos(\theta_2-\alpha)\sin(\theta_2-\alpha)\right]}{2} \\ \dfrac{(-\kappa_x+\kappa_y)\left[\cos(\theta_1-\alpha)\sin(\theta_1-\alpha)+\cos(\theta_2-\alpha)\sin(\theta_2-\alpha)\right]}{2} & \dfrac{\kappa_x\left[\sin^2(\theta_1-\alpha)+\sin^2(\theta_2-\alpha)\right]+\kappa_y\left[\cos^2(\theta_1-\alpha)+\cos^2(\theta_2-\alpha)\right]}{2} \end{pmatrix} \tag{S14}$$

In addition, the effective thermal conductivity κ^{eff} in cylindrical coordinates can be expressed in the following equation:

$$\kappa^{\text{eff}}=\begin{pmatrix} \kappa_{rr}^{\text{eff}} & \kappa_{r\theta}^{\text{eff}} \\ \kappa_{\theta r}^{\text{eff}} & \kappa_{\theta\theta}^{\text{eff}} \end{pmatrix}=\begin{pmatrix} \dfrac{\partial x}{\partial r} & \dfrac{\partial x}{\partial \theta} \\ \dfrac{\partial y}{\partial r} & \dfrac{\partial y}{\partial \theta} \end{pmatrix}\begin{pmatrix} \kappa_{xx}^{\text{eff}} & \kappa_{xy}^{\text{eff}} \\ \kappa_{yx}^{\text{eff}} & \kappa_{yy}^{\text{eff}} \end{pmatrix}\begin{pmatrix} \dfrac{\partial x}{\partial r} & \dfrac{\partial y}{\partial r} \\ \dfrac{\partial x}{\partial \theta} & \dfrac{\partial y}{\partial \theta} \end{pmatrix} \tag{S15}$$

Considering the constant temperature gradient ∇T_x along the negative direction of the y axis, the heat flux can be shown as follows:

$$\begin{pmatrix} q_x \\ q_y \end{pmatrix}=-\begin{pmatrix} \kappa_{xx}^{\text{eff}} & \kappa_{xy}^{\text{eff}} \\ \kappa_{yx}^{\text{eff}} & \kappa_{yy}^{\text{eff}} \end{pmatrix}\begin{pmatrix} \partial T/\partial x \\ \partial T/\partial y \end{pmatrix} \tag{S16}$$

Then, the heat flux bending angle $\phi(\theta_1,\theta_2,\alpha)$ in the twisted interface can be expressed as follows:

$$\begin{aligned}\phi(\theta_1,\theta_2,\alpha)&=\tan^{-1}\left(\frac{\kappa_{yx}^{\text{eff}}}{\kappa_{xx}^{\text{eff}}}\right)\\ &=\tan^{-1}\left(\frac{(-\kappa_x+\kappa_y)\left[\cos(\theta_1-\alpha)\sin(\theta_1-\alpha)+\cos(\theta_2-\alpha)\sin(\theta_2-\alpha)\right]}{\kappa_x\left[\cos^2(\theta_1-\alpha)+\cos^2(\theta_2-\alpha)\right]+\kappa_y\left[\sin^2(\theta_1-\alpha)+\sin^2(\theta_2-\alpha)\right]}\right)\end{aligned} \tag{S17}$$

If $u=\left(\dfrac{\kappa_{\text{I}}}{\kappa_{\text{II}}}\right)w_{\text{I}}^2+\left(\dfrac{\kappa_{\text{I}}}{\kappa_{\text{II}}}\right)^2 w_{\text{I}}w_{\text{II}}+w_{\text{I}}w_{\text{II}}+\left(\dfrac{\kappa_{\text{I}}}{\kappa_{\text{II}}}\right)w_{\text{II}}^2$ and $v=(w_{\text{I}}+w_{\text{II}})^2\left(\dfrac{\kappa_{\text{I}}}{\kappa_{\text{II}}}\right)$, then we can simplify the heat flux bending angle $\phi(\theta_1,\theta_2,\alpha)$ shown below:

$$\phi(\theta_1,\theta_2,\alpha)=\tan^{-1}\left(\frac{(u-v)\left[\cos(\theta_1-\alpha)\sin(\theta_1-\alpha)+\cos(\theta_2-\alpha)\sin(\theta_2-\alpha)\right]}{u\left[\sin^2(\theta_1-\alpha)+\sin^2(\theta_2-\alpha)\right]+v\left[\cos^2(\theta_1-\alpha)+\cos^2(\theta_2-\alpha)\right]}\right) \tag{S18}$$

Supplementary Note 3: Theoretical reconfiguration of thermal magic angle

Here, based on the twisted thermotics theory (detailed in Supplementary Note 2), we manipulate the line-type moiré patterns to adjust the effective thermal conductivity tensor and customize thermal coupling with respect to the angle. When twisting the moiré patterns, the theoretical description of the thermal conductivity tensor between the two stripe layers is

provided in Eq. (S14).

Heat diffusion, distinct from photon dispersion and electron band structure, is intrinsically absent of a clearly defined magic angle, making it elusive to imagine a thermal analogue. By actively tuning the conductivity tensor within a twisted diffusion system, we seek to discover and design an analog thermal magic angle.

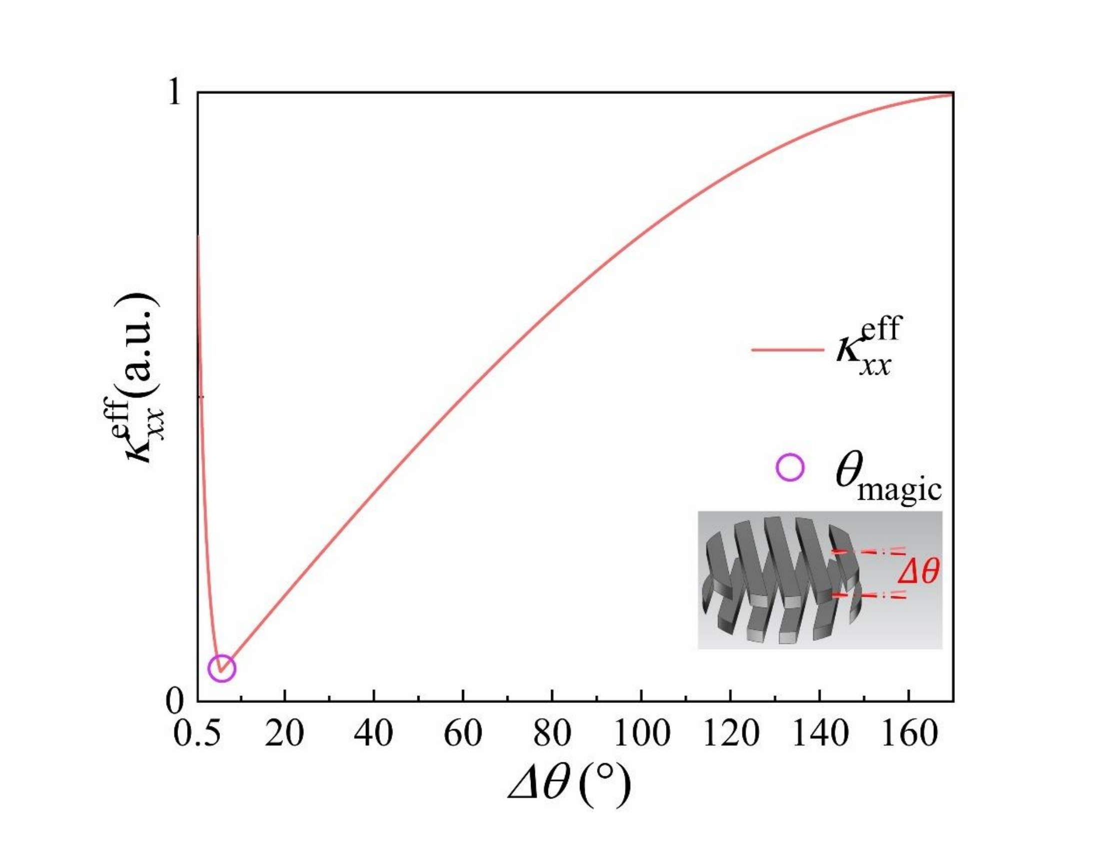

Fig. S2 Theoretical result of effective thermal conductivity $\kappa_{xx}^{\mathrm{eff}}$ (a.u. is short for arbitrary unit) along the x direction related to the twisted angle difference of two twisted stripe layers calculated by Eq. (S19), from which one could observe the thermal magic angle.

Thus, based on the related literatures[4,5], if the stripes with the same thermal conductivity are alternately arranged on two layers and the width of stripes should meet the required condition ($w_{\mathrm{I}} \geq w_{\mathrm{II}}$), as shown in Fig. S2, we can acquire a counterintuitive and different thermal conductivity $\kappa_{xx}^{\mathrm{eff}}$ along the uniaxial direction. Thus, the κ^{eff} can be expressed as

$$\kappa^{\mathrm{eff}}=\begin{pmatrix}\kappa_{xx}^{\mathrm{eff}} & \kappa_{xy}^{\mathrm{eff}}\\ \kappa_{yx}^{\mathrm{eff}} & \kappa_{yy}^{\mathrm{eff}}\end{pmatrix}$$

$$=\begin{cases}\begin{pmatrix}\dfrac{(w_{\mathrm{I}}+w_{\mathrm{II}})(\kappa_{\mathrm{II}}+\zeta_{\mathrm{I}}\kappa_{\mathrm{I}})+\zeta_{\mathrm{I}}(\kappa_{\mathrm{II}}-\kappa_{\mathrm{I}})w_{\mathrm{II}}}{(w_{\mathrm{I}}+w_{\mathrm{II}})(\kappa_{\mathrm{II}}+\zeta_{\mathrm{I}}\kappa_{\mathrm{I}})-(\kappa_{\mathrm{II}}-\kappa_{\mathrm{I}})w_{\mathrm{II}}} & 0\\ 0 & \dfrac{w_{\mathrm{I}}\kappa_{\mathrm{I}}+w_{\mathrm{II}}\kappa_{\mathrm{II}}}{w_{\mathrm{I}}+w_{\mathrm{II}}}\end{pmatrix} & (\theta_1=\theta_2=\dfrac{m\pi}{2})\\ \begin{pmatrix}\dfrac{\kappa_x\left[\cos^2(\theta_1-\alpha)+\cos^2(\theta_2-\alpha)\right]+\kappa_y\left[\sin^2(\theta_1-\alpha)+\sin^2(\theta_2-\alpha)\right]}{2} & \dfrac{(-\kappa_x+\kappa_y)\left[\cos(\theta_1-\alpha)\sin(\theta_1-\theta)+\cos(\theta_2-\alpha)\sin(\theta_2-\alpha)\right]}{2}\\ \dfrac{(-\kappa_x+\kappa_y)\left[\cos(\theta_1-\alpha)\sin(\theta_1-\alpha)+\cos(\theta_2-\alpha)\sin(\theta_2-\alpha)\right]}{2} & \dfrac{\kappa_x\left[\sin^2(\theta_1-\alpha)+\sin^2(\theta_2-\alpha)\right]+\kappa_y\left[\cos^2(\theta_1-\alpha)+\cos^2(\theta_2-\alpha)\right]}{2}\end{pmatrix} & (\theta_1-\theta_2\neq m\pi)\end{cases}\quad\text{(S19)}$$

where d is the thickness of stripes in either upper or lower layer and $\zeta_{\mathrm{I}}=\sqrt{3}\log(w_{\mathrm{I}}/d)$. The subscripts $_{\mathrm{I}}$ and $_{\mathrm{II}}$ indicate the two kinds of stripe in the upper and lower layers, respectively. The subscripts $_1$ and $_2$ indicate upper and lower layers, respectively. The subscripts $_x$ and $_y$ indicate the x direction and y direction, respectively.

In fact, from the above Eq. (S19), if neglecting the influence of the structure parameters, we can find out the value of the anisotropic thermal conductivity tensor can be discontinuous at $\theta_1=\theta_2=m\pi/2$ (m = 0,1, . . ., n) for not large enough stripes. The anisotropic thermal conductivity tensor should continuously change via twisted manipulations, and thus the influence of the structure parameters should be considered. Meanwhile, considering the practical applications for a twisted diffusion system, the zone with stripes cannot be infinitely large and the influence of the structure parameter should be further investigated. Moreover, from the simulation results of Fig. 2c in the main text, the influence of the structure parameters induces a gradient effect from a maximum value at the original angles to a minimum value after twisting a thermal magic angle. Thus, we need to find a theoretical relation between the minimum value and the thermal magic angle accurately.

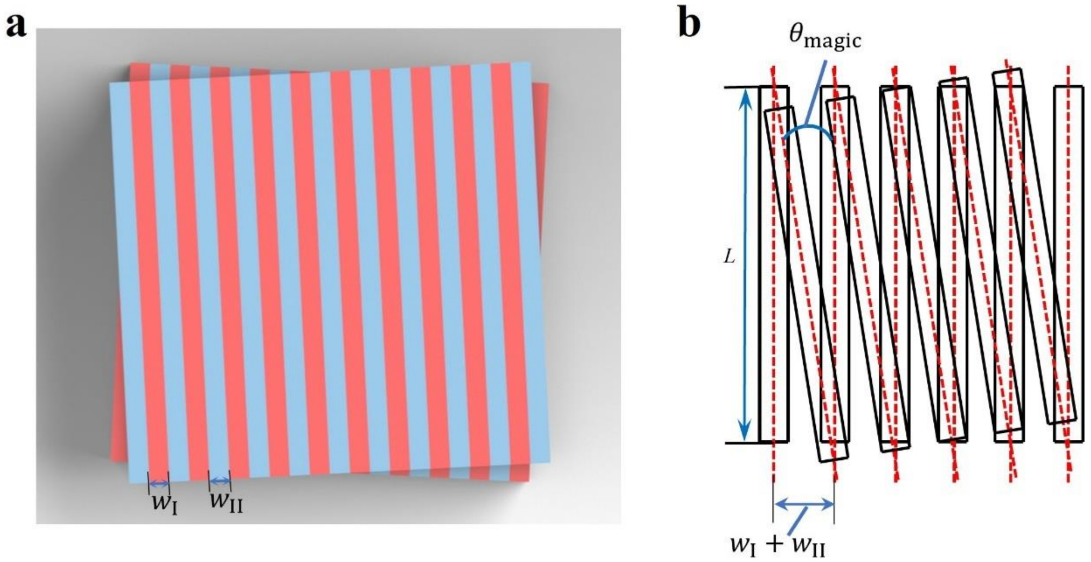

Fig. S3 Schematics of the zigzag connection at two layers. a 3D Schematic of the stripes with a zigzag connection. Red stripes (I) possess a higher thermal conductivity and blue stripes (II) have a lower thermal conductivity. **b** 2D Schematic of the stripes with a zigzag connection.

As shown in Fig. S3, when the direction of heat flux is along the positive direction of *x* axis, it is easy to find out that twisting the stripes to form a zigzag structure can maximumly reduce heat diffusion. Specifically, Fig. S3a provides a 3D schematic of the stripes with a zigzag connection while Fig. S3b describes the corresponding 2D schematic of the stripes with a zigzag connection. Thus, the current form of theoretical reconfiguration of the thermal magic angle is described as follows:

$$\theta_{\mathrm{magic}}\big|_{\mathrm{rec}} \approx \arcsin\left(\frac{w_{\mathrm{I}} + w_{\mathrm{II}}}{L}\right) \tag{S20}$$

where L is the length of two different stripes in a rectangular region. The subscript $_{\mathrm{rec}}$ indicates the rectangular region. The subscripts $_{\mathrm{I}}$ and $_{\mathrm{II}}$ indicate the two kinds of stripes in each layer, respectively.

For simplicity, the width of blue stripes and red stripes in each layer (upper or lower) is set to be equal and thus w can be used to define the width of two kinds of stripes now. Then, for the stripes in a circular region, we can assume the blue stripes are changed to be the air with a lower thermal conductivity and the red stripes are maintained as illustrated in Fig. S4a.

However, we can easily observe that the lengths of the stripes in a circular region are not uniform. Thus, if we want to strictly obtain a more theoretical derivation, we need to transform the stripes in a circular region into effective stripes with a uniform length as shown in Fig. S4b. Therefore, we need to calculate the average length of the stripes as below:

$$\langle L \rangle = \frac{\sum_{n=1}^{N} 2R_1 \sin\beta_n}{N} = \frac{\int_0^{\pi} 2R_1 \sin\eta d\eta}{\int_0^{\pi} d\eta} = \frac{4}{\pi} R_1 \tag{S21}$$

$$\langle L \rangle = \frac{2w}{\sin\theta_{\text{magic}}} \Rightarrow \sin\theta_{\text{magic}} = \frac{2w}{\langle L \rangle} = \frac{2w}{\frac{4}{\pi} R_1} = \frac{\pi w}{2R_1} \tag{S22}$$

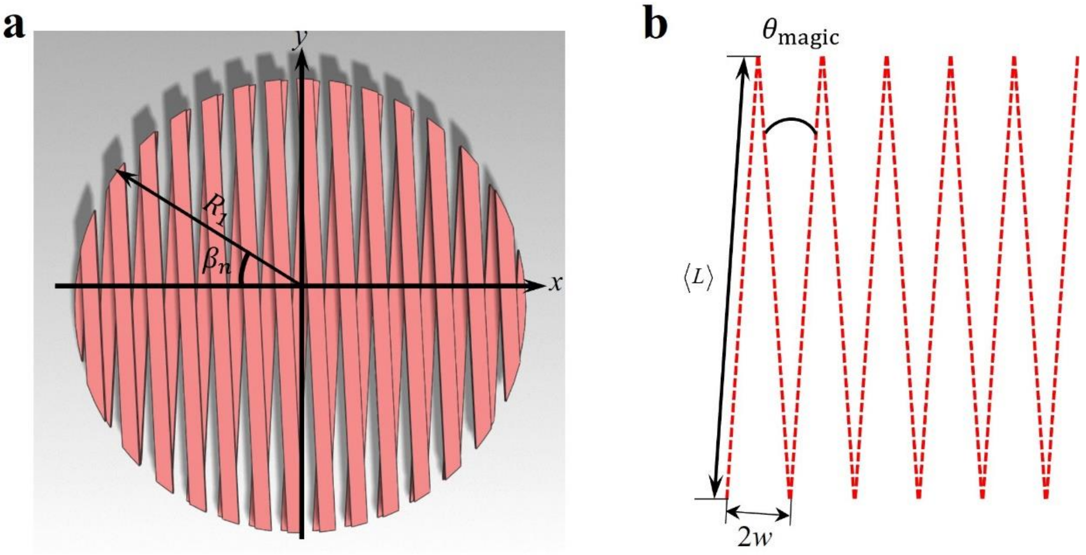

Fig. S4 Theoretical derivation of the thermal magic angle. a Schematic of the stripes in a circular region. **b** Schematic of the effective stripes.

More interestingly, for the two-line moiré patterns, we can observe that there is a rhombus with four sides in a cell of the lattice as shown in Fig. S5 and it is formed with the following relation as follows:

$$d = \frac{p}{\sin\alpha} \tag{S23}$$

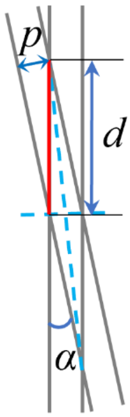

Fig. S5 Schematics of a rhombus in a cell of the lattice for the two-line moiré patterns.

Herein, in contrast to a rhombus in a cell of the lattice for two-line moiré patterns, we can observe the following mapping for the thermal magic angle under the zigzag condition:

$$d \to \langle L \rangle, p \to 2w, \theta_{\text{magic}} \to \alpha \tag{S24}$$

Eventually, for the above stripes in a circular region, we can achieve the theoretical reconfiguration of the thermal magic angle as shown below:

$$\theta_{\text{magic}} = \arcsin(\frac{\pi w}{2R_1}) \tag{S25}$$

To construct a magic angle phenomenon in a twisted diffusion system, the zigzag connection in the two layers should be maintained and it is easy to observe that the least number of stripes in each layer is three.

Supplementary Note 4: Extended theoretical principle in twisted metadevices related to the thermal magic angle phenomenon

As we know, the heat conduction equation at steady states can be expressed as[1]:

$$\nabla \cdot (-\kappa \nabla T) = 0 \tag{S26}$$

Then, for the anisotropic thermal conductivity medium, the governing equation can be expanded in cylindrical coordinates as shown below:

$$\frac{\partial}{\partial r}\left(r\kappa_{rr} \frac{\partial T}{\partial r} \right) + \frac{\partial}{\partial \theta}\left(\kappa_{\theta\theta} \frac{\partial T}{r\partial \theta} \right) = 0 \tag{S27}$$

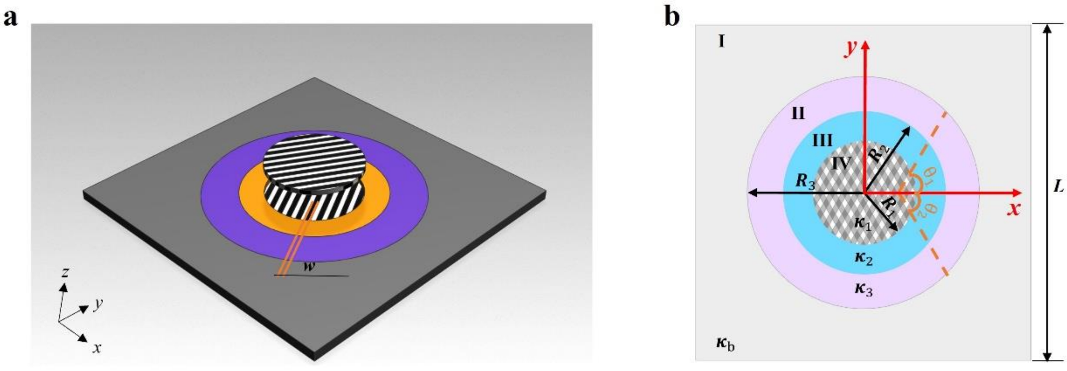

Fig. S6 Functional regions of the structure in a twisted diffusion system. (a) The 3D schematic of the functional regions in a twisted diffusion system. (b) The 2D schematic of the functional regions in a twisted diffusion system.

Now, as shown in Fig. S6, there is just a twisted bilayer system. The subscripts $_1$, $_2$, $_3$ and $_4$ except the radii (R_1 , R_2 , and R_3) and the thermal conductivities (κ_1^{eff} , κ_2^{eff} , and κ_3) indicate the four regions I, II, III and IV in this system, respectively. The effective thermal conductivities of the four regions I, II, III and IV are denoted by κ_b , κ_3 , κ_2^{eff} , and κ_1^{eff} , respectively. Meanwhile, the 3D model of this structure system and 2D functional regions in this structure system are illustrated in Fig. S6a and Fig. S6b, respectively. Then, we can obtain the general solutions of this system just as follows:

$$\begin{cases} T_1 = \left(A_1 r + B_1 r^{-1}\right)\cos\theta & \left(r > R_3\right) \\ T_2 = \left(A_2 r + B_2 r^{-1}\right)\cos\theta & \left(R_2 < r \le R_3\right) \\ T_3 = \left(A_3 r + B_3 r^{-1}\right)\cos\theta & \left(R_1 < r \le R_2\right) \\ T_4 = \left(A_4 r + B_4 r^{-1}\right)\cos\theta & \left(r \le R_1\right) \end{cases} \tag{S28}$$

where A and B with the subscripts $_1$, $_2$, $_3$ and $_4$ are the unknown parameters that can be determined by boundary conditions, respectively.

Therefore, the boundary conditions at these regional interfaces can be described as:

$$
\begin{cases}
T_1\big|_{r=R_3} = T_2\big|_{r=R_3} \\
\kappa_{\mathrm{b}} \dfrac{\partial T_1}{\partial r}\bigg|_{r=R_3} = \kappa_3 \dfrac{\partial T_2}{\partial r}\bigg|_{r=R_3} ; \\
T_2\big|_{r=R_2} = T_3\big|_{r=R_2} \\
\kappa_3 \dfrac{\partial T_2}{\partial r}\bigg|_{r=R_2} = \kappa_2^{\mathrm{eff}} \dfrac{\partial T_3}{\partial r}\bigg|_{r=R_2} ; \\
T_3\big|_{r=R_1} = T_4\big|_{r=R_1} \\
\kappa_2^{\mathrm{eff}} \dfrac{\partial T_3}{\partial r}\bigg|_{r=R_1} = \kappa_{1,xx}^{\mathrm{eff}} \dfrac{\partial T_4}{\partial r}\bigg|_{r=R_1} .
\end{cases}
\tag{S29}
$$

Combining these Eqs. (S28-29), the matching functions can be written as:

$$
\frac{\kappa_{\mathrm{b}} + \kappa_3}{-\kappa_{\mathrm{b}} + \kappa_3} \cdot \frac{R_2^2}{R_3^2} = \frac{\left[\dfrac{\left(\kappa_2^{\mathrm{eff}} + \kappa_{1,xx}^{\mathrm{eff}}\right)}{\left(\kappa_2^{\mathrm{eff}} - \kappa_{1,xx}^{\mathrm{eff}}\right)} \cdot \dfrac{R_2^2}{R_1^2} + 1 \right] + \dfrac{\kappa_2^{\mathrm{eff}}}{\kappa_3} \left[\dfrac{\left(\kappa_2^{\mathrm{eff}} + \kappa_{1,xx}^{\mathrm{eff}}\right)}{\left(\kappa_2^{\mathrm{eff}} - \kappa_{1,xx}^{\mathrm{eff}}\right)} \cdot \dfrac{R_2^2}{R_1^2} - 1 \right]}{\left[\dfrac{\left(\kappa_2^{\mathrm{eff}} + \kappa_{1,xx}^{\mathrm{eff}}\right)}{\left(\kappa_2^{\mathrm{eff}} - \kappa_{1,xx}^{\mathrm{eff}}\right)} \cdot \dfrac{R_2^2}{R_1^2} + 1 \right] - \dfrac{\kappa_2^{\mathrm{eff}}}{\kappa_3} \left[\dfrac{\left(\kappa_2^{\mathrm{eff}} + \kappa_{1,xx}^{\mathrm{eff}}\right)}{\left(\kappa_2^{\mathrm{eff}} - \kappa_{1,xx}^{\mathrm{eff}}\right)} \cdot \dfrac{R_2^2}{R_1^2} - 1 \right]}
\tag{S30}
$$

If $\kappa_2^{\mathrm{eff}} \to 0$, we can simplify the matching function Eq. (S30) as shown in the following equation:

$$
\frac{\kappa_{\mathrm{b}} + \kappa_3}{-\kappa_{\mathrm{b}} + \kappa_3} \cdot \frac{R_2^2}{R_3^2} = 1 \Rightarrow \kappa_3 = \kappa_{\mathrm{b}} \frac{R_3^2 + R_2^2}{R_3^2 - R_2^2}
\tag{S31}
$$

Then, for the current case with the upper and lower layers in region IV, each layer (upper or lower) in the central region is composed of evenly spaced stripes with stripe width *w*, stripe thickness *d* and different thermal conductivities κ_{I} and κ_{II} . The anisotropic thermal conductivity tensors for two layers can be derived based on equations S11 and S12.

The effective thermal conductivity tensor can be achieved at the interface of the upper and lower layers after twisting the upper layer an angle θ_1 and the lower layer an angle θ_2, and we can obtain the expression of this equation in the Cartesian coordinate based on equations S19.

Then, the heat flux bending angle $\phi_1(\theta_1, \theta_2, \alpha)$ can be expressed as follows:

$$
\begin{aligned}
\phi_1(\theta_1, \theta_2, \alpha) &= \tan^{-1}\left(\frac{\kappa_{1,yx}^{\mathrm{eff}}}{\kappa_{1,xx}^{\mathrm{eff}}} \right) \\
&= \tan^{-1}\left(\frac{\left(-\kappa_x + \kappa_y\right)\left[\cos(\theta_1 - \alpha)\sin(\theta_1 - \alpha) + \cos(\theta_2 - \alpha)\sin(\theta_2 - \alpha)\right]}{\kappa_x\left[\cos^2(\theta_1 - \alpha) + \cos^2(\theta_2 - \alpha)\right] + \kappa_y\left[\sin^2(\theta_1 - \alpha) + \sin^2(\theta_2 - \alpha)\right]} \right)
\end{aligned}
\tag{S32}
$$

Assuming $a=\left(\frac{\kappa_{\mathrm{I}}}{\kappa_{\mathrm{II}}}+1\right)^2 w^2$ and $b=4w^2\frac{\kappa_{\mathrm{I}}}{\kappa_{\mathrm{II}}}$, then we can simplify the heat flux bending angle $\phi_1(\theta_1,\theta_2,\alpha)$ shown below:

$$\phi(\theta_1,\theta_2,\alpha)=\tan^{-1}\left(\frac{(a-b)\left[\cos(\theta_1-\alpha)\sin(\theta_1-\alpha)+\cos(\theta_2-\alpha)\sin(\theta_2-\alpha)\right]}{a\left[\sin^2(\theta_1-\alpha)+\sin^2(\theta_2-\alpha)\right]+b\left[\cos^2(\theta_1-\alpha)+\cos^2(\theta_2-\alpha)\right]}\right) \tag{S33}$$

Then, assuming that region III is composed of the aluminum alloy and air with two different volume ratios f_{m} and f_{air} with the corresponding thermal conductivities κ_{m} and κ_{air}, the effective thermal conductivity κ_2^{eff} is then:

$$\kappa_2^{\mathrm{eff}}=f_{\mathrm{m}}\kappa_{\mathrm{m}}+f_{\mathrm{air}}\kappa_{\mathrm{air}} \tag{S34}$$

For the twisted metadevice shown in Fig. S6, the temperature gradient $\frac{\partial T_4}{\partial x}$ in the central stripe region can be written as follows:

$$\frac{\partial T_4}{\partial x}=\frac{4}{\left[1+\left(\frac{R_2}{R_3}\right)^2\right]\bullet\left\{\left[1-\left(\frac{R_1}{R_2}\right)^2\right]\frac{\kappa_{1,xx}^{\mathrm{eff}}}{\kappa_2^{\mathrm{eff}}}+\left[1+\left(\frac{R_1}{R_2}\right)^2\right]\right\}}\bullet\frac{T_{\max}-T_{\min}}{L} \tag{S35}$$

More importantly, considering the influence of the stripe width *w* in the circular region IV as shown in Fig. S6, it is easy to find out that the formation of a zigzag structure in a circular region (Fig. S4) can maximumly reduce the heat diffusion via twisting a thermal magic angle $\theta_{\mathrm{magic}}=\arcsin(\pi w/2R_1)$ just as the above Eq. (S25), resulting in the function switching from cloaking to concentration.

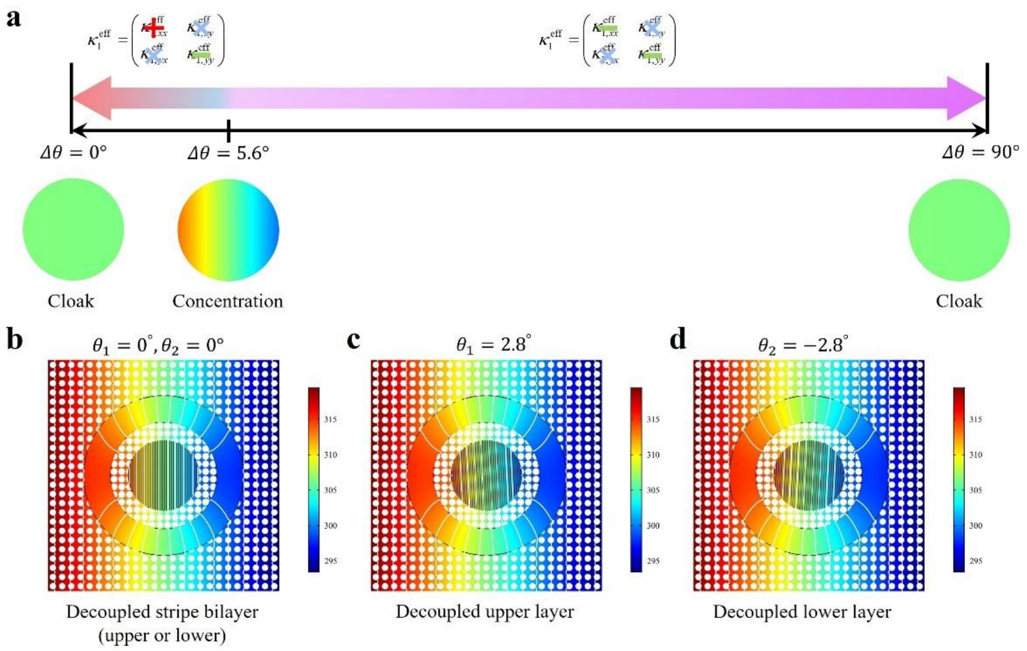

Fig. S7 The corresponding coupled and decoupled phenomena with respect to the twist angle. a The coupling change with respect to the twist angle **b, c, d** The corresponding phenomena of decoupled stripe bilayer with respect to several special twist angles. The $+$, $\times$, and $-$ refer to enhancement, counteraction and almost unchangeableness of the corresponding coupling strength, respectively.

Eventually, as shown in Fig. S7a, we have reconstructed a strong coupling at the original state with zero degrees via a dislocation arrangement of stripes at two layers, and it can couple the main diagonal item $\kappa_{1,xx}^{\text{eff}}$, the off-diagonal items $\kappa_{1,xy}^{\text{eff}}$ and $\kappa_{1,yx}^{\text{eff}}$ in the anisotropic thermal conductivity tensor κ_{1}^{eff} through a symmetric twisted manipulation from zero degrees to the analog thermal magic angle. After surpassing the thermal magic angle, the coupling in the main diagonal item $\kappa_{1,xx}^{\text{eff}}$ mainly vanishes due to the almost same change of the $\kappa_{1,xx}^{\text{eff}}$ in only one stripe layer without coupling and the coupling in the off-diagonal items of the anisotropic thermal conductivity tensor κ_{1}^{eff} is still maintained due to the left or right heat flux rotation in one uncoupled stripe layer being cancelled out through a symmetric twisted manipulation. As a result, it is easy to observe a cloaking effect (Fig. 3b) at zero degrees in the coupled stripe bilayer while we can only acquire thermal concentration in two decoupled stripe layers (Fig.

S7b), indicating the existence of a strong coupling. When it comes to the thermal magic angle, the coupling in its off-diagonal items is still maintained at least, leading to a thermal concentration (Fig. 3c) without the left or right rotation effect (Fig. S7c and S7d).

Supplementary Note 5: The photographs of experimental setups

The photographs of these actual experimental setups for twisted thermal metadevice based on twisted thermotics are shown in Fig. S8.

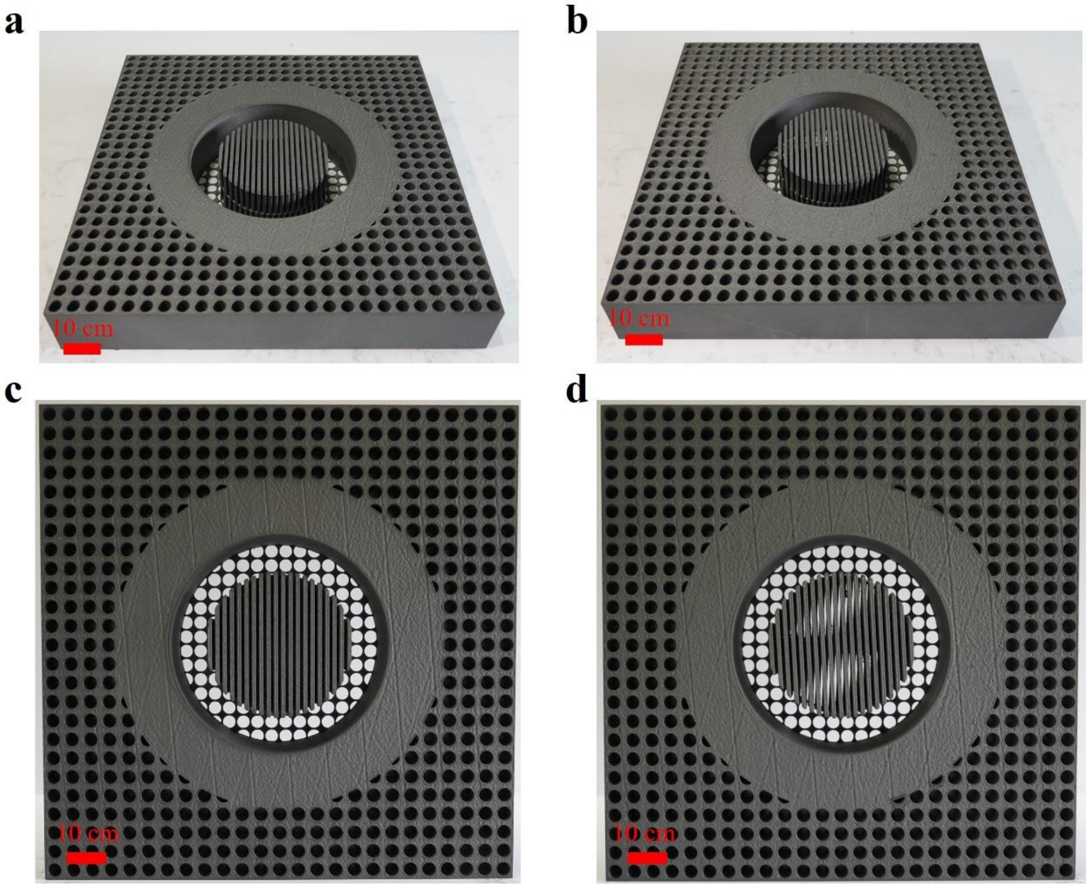

Fig. S8 The photograph of the experimental setup. a, c Photographs of the oblique view (a) and top view (c) of the experimental setup with zero degrees. **b, d** Photographs of the oblique view (b) and top view (d) of the experimental setup with the thermal magic angle.

Supplementary Note 6: The evolutions of thermal magic angle phenomena in twisted diffusion systems from two layers to three layers

To further investigate the influence of twisted layer number on the thermal magic angle phenomenon, a twisted trilayer diffusive system has been numerically carried out and the

evolutions of temperature distribution and gradient are shown in Fig. S9.

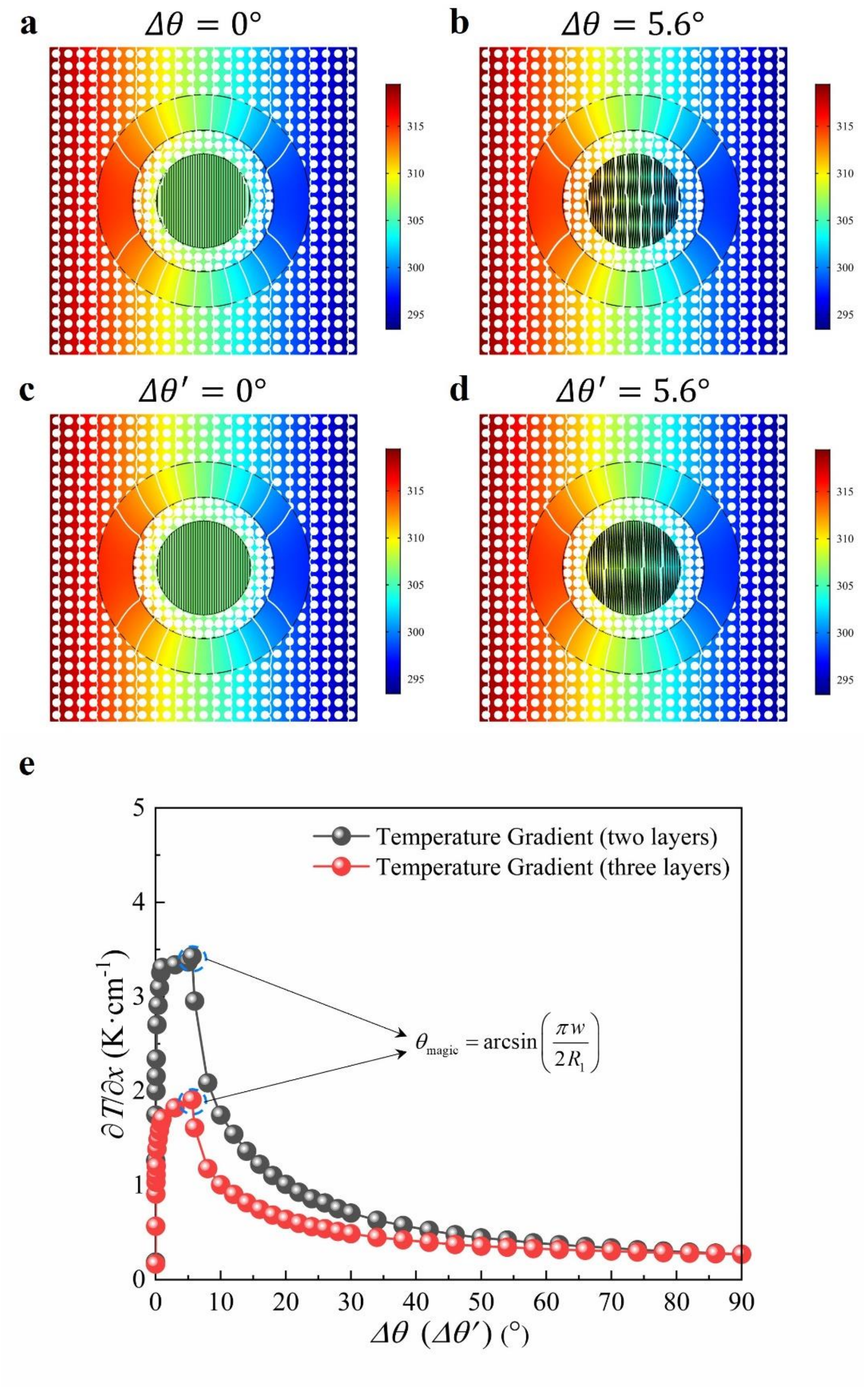

Fig. S9 Temperature profiles of twisted diffusion systems with two and three layers for two different combinations of twisted angles. a, b, c, d Temperature distributions of thermal cloak-to-concentration via twisting a magic angle ($\Delta\theta = \theta_1 - \theta_2$, $\Delta\theta' = \theta_1 - \theta_2 + \theta_3$ and $\theta_3 = 0$). **e** The evolutions of temperature gradient in twisted diffusion systems with two and three layers.

Supplementary Note 7: Demonstration of the thermal magic angle phenomenon under an elliptic boundary condition.

Considering there exists an elliptical boundary with a shell of inner and outer boundaries ξ_1 and ξ_2, we can obtain the heat conduction equation in the elliptic coordinate (ξ,θ) as

$$\nabla^2 T = \frac{1}{\rho^2}\left(\frac{\partial^2 T}{\partial \xi^2}+\frac{\partial^2 T}{\partial \theta^2}\right)=0 \tag{S36}$$

Then, according to some similar derivations in Supplementary Note 4 and other related reference[6], when $\kappa_2^{\text{eff}} \to 0$, the matching functions can be derived as

$$\kappa_{\text{b}} = \begin{pmatrix} \kappa_3 \coth\xi_2 \tanh(\xi_2-\xi_1) & 0 \\ 0 & \kappa_3 \tanh\xi_2 \tanh(\xi_2-\xi_1) \end{pmatrix} \tag{S37}$$

where κ_{b} and κ_3 are the thermal conductivity of background and region II, respectively.

Then, based on the above derived matching function, we can realize the functional switching from cloaking to concentration under an elliptic boundary condition, which makes it possible to generalize our approach to non-uniform boundary conditions as shown in Fig. S10. We believe the cases in non-uniform boundary conditions and for more complicated geometries might exist more interesting phenomena and look forward to future works on these topics.

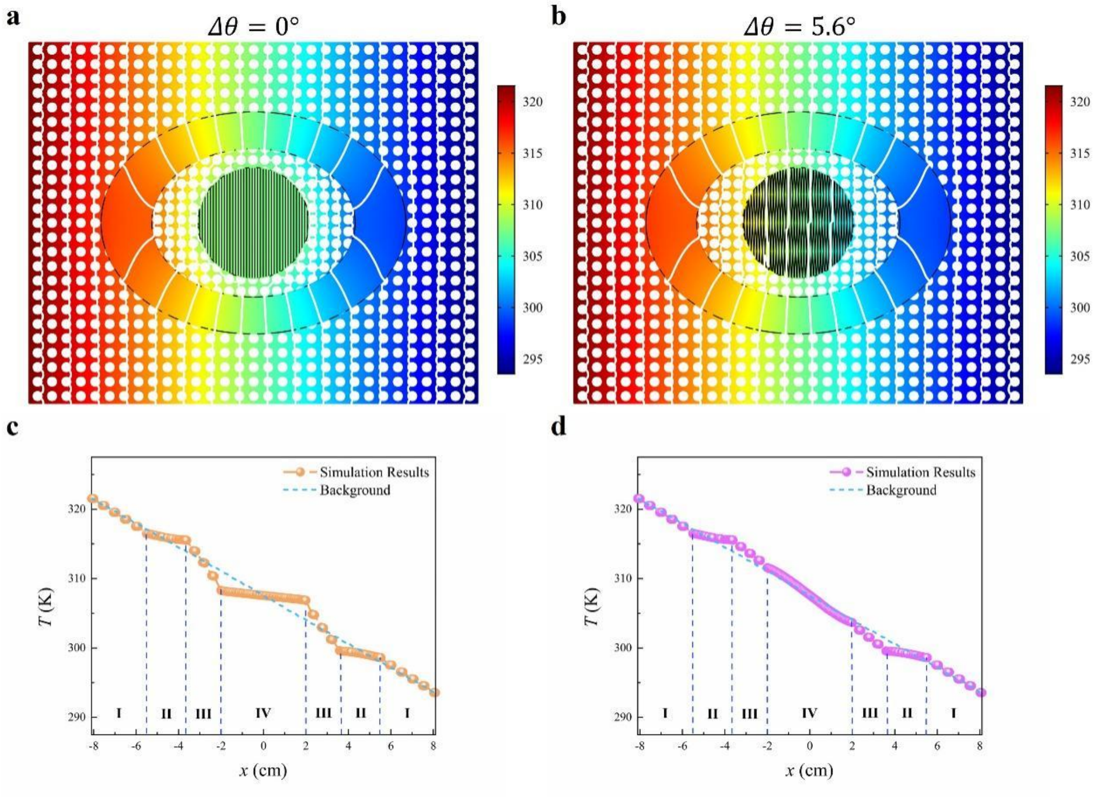

Fig. S10 Numerical demonstration of the thermal magic angle phenomenon under an elliptic boundary condition. a, b Temperature distributions of thermal cloak-to-concentration via twisting a magic angle. **c, d** Temperature distributions along the line (y = 0) in twisted diffusion systems under an elliptic boundary condition.

Supplementary Note 8: Demonstration of the effect of the graphene coatings on thermal magic angle phenomenon in a twisted system.

Since the introduction of the graphene layer with a thickness of d_{gra} in the system, the interlayer coupling can be affected by the thermal resistance between the graphene and aluminum alloy. In the study of thermal transport in materials, the interlayer coupling of the effective thermal conductivity tensor plays a crucial role in understanding and controlling heat flux. By considering this new interlayer coupling, we can explore more complex thermal fields, enabling us to manipulate heat transfer with greater precision. Additionally, the introduction of graphene coatings in our twisted bilayer diffusive systems offers an exciting opportunity to expedite the formation of steady-state thermal coupling phenomena, as demonstrated in Fig. S11.

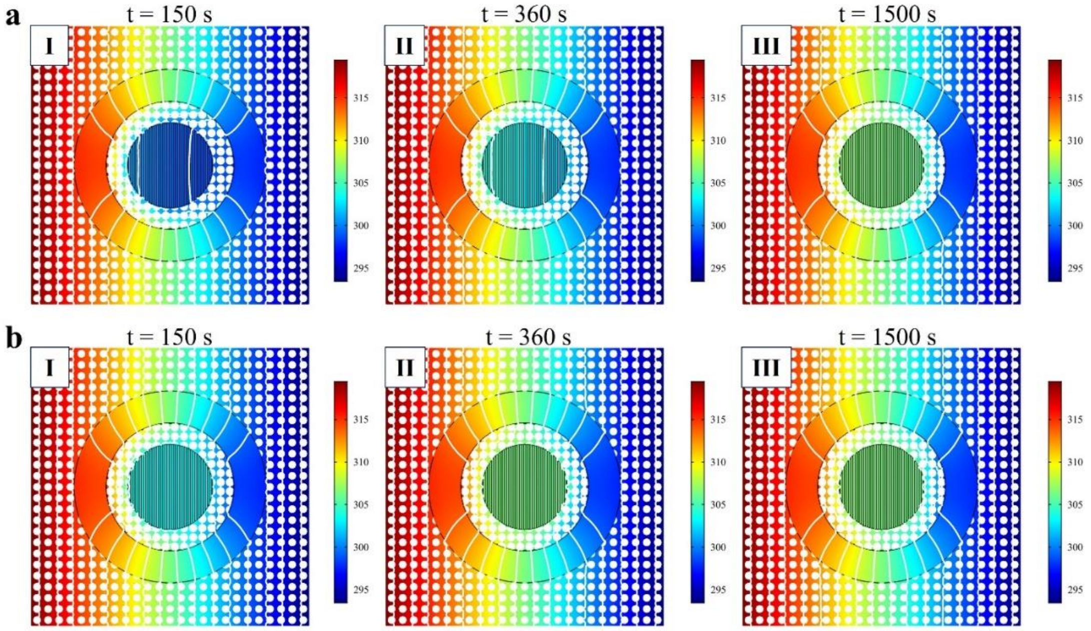

Fig. S11 Transient evolutions a (without graphene coatings) and b (with graphene coatings) of thermal cloak in our twisted diffusive systems at original twisted angles.

References

1. Li, Y. *et al.* Transforming heat transfer with thermal metamaterials and devices. *Nat. Rev. Mater.* **6**, 488–507 (2021).
2. Yang, T., Vemuri, K. P. & Bandaru, P. R. Experimental evidence for the bending of heat flux in a thermal metamaterial. *Appl. Phys. Lett.* **105**, 083908 (2014).
3. Su, Y. *et al.* Path-dependent thermal metadevice beyond Janus functionalities. *Adv. Mater.* **33**, 2003084 (2021).
4. Progelhof, R. C., Throne, J. L. & Ruetsch, R. R. Methods for predicting the thermal conductivity of composite systems: A review. *Polym. Eng. Sci.* **16**, 615–625 (1976).
5. Agari, Y. & Uno, T. Estimation on thermal conductivities of filled polymers. *J. Appl. Polym. Sci.* **32**, 5705–5712 (1986).
6. Han, T. *et al.* Full-parameter omnidirectional thermal metadevices of anisotropic geometry. *Adv. Mater.* **30**, 1804019 (2018).
7. Lambropoulos, J. C. et al. Thermal conductivity of dielectric thin films. *J. Appl. Phys.* 66, 4230–4242 (1989).
